# Medical Department of the University of Buffalo

**Published:** 1852-08

**Authors:** 


### EDITORIAL DEPARTMENT.

---

*Medical Department of the University of Buffalo, Session of* 1852–3.—On reference to the advertisement (see advertising sheet, cover) it will be perceived that the Faculty of this institution have made some changes in the commencement of the terms, the fees, etc., for the Session of 1852–3. The preliminary term, devoted mainly to the study of practical anatomy, and hospital instruction, will commence, for the next session, on the *fourth Wednesday* (24*th*) *of November*, and continue to the opening of the regular term. The regular term will commence on the *first Wednesday* (5*th*) *January*, 1853, and continue for the usual period, viz., four months. This change of time has been deemed advisable for the present collegiate year, and it is believed that it will meet the convenience and wishes of medical students, as well as, if not better than the former arrangement. A considerable number of students heretofore have found it difficult to attend so early as the month of October, or even to enter at the beginning of the regular term in the early part of November. The postponement adopted for the present year will accommodate those who are unable to come at an earlier date, and will afford advantages to those with whom the time of commencement is a matter of indifference, not less than if no change of time had been made.

In view of the course pursued by other institutions, with which, from their geographical situation, the school at Buffalo is necessarily brought into competition, the Faculty have resolved to adopt, for the next session, a change with respect to fees. The aggregate fees for the tickets of the seven professors will be *fifty dollars;* and in particular cases, a note, satisfactorily endorsed, upon interest, will be received. The latter new feature in the management of the institution is adopted, for the present collegiate year, for the same reason that the reduction in fees has been made.

The only change in the members of the Faculty that has taken place, is the resignation of Prof. Palmer, in consequence of his having accepted an appointment in the University of Louisville, and his engagements there and at Woodstock being such as to prevent his giving the anatomical course in the school at Buffalo. The well known ability of Prof. P., and his high reputation as a teacher, render the necessity of his resignation greatly to be regretted. The Faculty, however, have been fortunate in securing the services of Prof. E. M. Moore in that important branch of medical instruction. Prof. Moore has for many years been a teacher of anatomy and surgery, holding the latter chair in the medical institutions at Woodstock, Vt., and Pittsfield, Mass. His reputation and experience furnish an ample guaranty that the department of anatomy willl be thoroughly and efficiently taught.

Since the last session the subject of morbid anatomy has been added to the chair of Physiology, and will be taught by Prof. Dalton, the incumbent of the chair. Prof. Dalton, immediately after the close of the last session, sailed for Europe, and is passing the summer at Paris, prosecuting investigations relating to the departments of instructions assigned to his chair. He will return by the commencement of the regular term.

With the present organization of the school; the admirable opportunities for clinical hospital instruction which are placed at its disposal; the conveniences of the college edifice; facilities for practical anatomy, etc., there is every reason to look for its continued and increasing prosperity. So far as a judgment can be formed in anticipation, the prospect is that the class for the next session will be large.

It may be proper to add, that the professor of the principles and practice of medicine will give the course in that department during the next session. This notice seems to be required in consequence of the incumbent having accepted an appointment in the University of Louisville. The course will be given, at Buffalo, during the last half of the term.

